# The Treatment of Pulmonary Phthisis*Read before the Georgia Medical Association, April, 1891.

**Published:** 1891-06

**Authors:** Thomas D. Coleman

**Affiliations:** Augusta, Ga.


Southern Medical Record.

A MONHLY JOURNAL OF MEDICINE AND SURGERY.

Vol. XXI. Atlanta, Ga., June, 1891. No. 6.

### Original Articles.

### THE TREATMENT OF PULMONARY PHTHISIS.*

BY THOMAS D. COLEMAN, A. B., M. D., AUGUSTA, GA.

A better introduction to a carefully prepared paper on this subject cannot be given than the proposition of Graves as adduced by Weber, that "it would be of the greatest importance to know how an individual could be made phthisical, since by following out the opposite method we could prevent phthisis." This feeling of helplessness has at times been experienced by every earnest worker in the science and art of medicine. This cry for more light has been deep in the mouth of every conscientious groper after truth, and I rejoice to say that almost every nightfall brings out a new star in the firmament of medical endeavor and experience. People in general are only too prone to lay the charge of sluggishness at the door of the physician, because of the absence of tangible proof of work done. The malady which forms the subject of this paper is the many-headed monster which has vanquished one ambitious victim after another; and while a part of his power for evil has been destroyed, he still is very potent and awaits the coming of some medical Hercules, who will not only amputate his many heads, but will as rapidly sear over the stumps with his tubercle destroyer. If one thinks that this subject has not agitated the medical world, let him simply glance into the literature of the subject from the age of heathen philosophy down to

*Read before the Georgia Medical Association, April, 1891.

the present hour and he will find an amount of material that will surprise and at the same time appal him, if he must compass it. A quarter of a century ago it was estimated that "in the temperate zones, where the civilized inhabitants of the globe are located, one-tenth of the population died of this malady." Another, not less eminent authority, states that no less than one-seventh of the yearly mortality in this country is due to pulmonary phthisis. Aitken, in his "Science and Art of Medicine," says: "The history of phthisis in armies will at once show how materially the prevalence of such a disease influences the wealth and military strength of a nation. In Prussia, phthisis caused 27 per cent. of the total mortality; in Austria, 25 per cent.; in France, 22.9 per cent.; in Hanover, 39.4 per cent.; in Belgium, 30 per cent.; in Portugal, 22 per cent." In the United States' armies, during the first two years of the civil war, there were reported in the first year 2,508 cases, of whom 550 died of pulmonary phthisis; in the second year, 5,596, with 2,040 deaths—being a little more than eight cases per thousand of the mean strength for the first year and nine per thousand for the second. The deaths were 1 to 4 5 cases in the first year and 1 to every 2.7 during the second. The results in the British army during that period were more disastrous still.

In a table prepared by Dr. Parks it is shown that of male civilians in all England and Wales the death rate was greatest from the ages of 25 to 45, being in that period 4.02 per one thousand.

These facts, together with a completer and more sound knowledge of the ætiology and pathology of this trouble, makes the further investigation and study of this subject intensely absorbing. A disease which annually saps so much of the world's strength is a fit theme for discussion; and the recent investigations of that acute observer and scientist, Robert Koch, have set not only the medical world, but humanity at large, at a fever heat of expectancy. One finds the medical, and even the secular press, burdened with this theme.

PROPHYLACTIC TREATMENT.

In a disease which annually takes off so large a number of the human family, the means by which this unfortunate percentage can be made less, is an object of the very highest importance. The prophylactic treatment is by far the heaviest in the scale, and for obvious reasons yields the happiest results. To this side of the question, too much attention cannot be paid. If the contagion can be prevented the disease will be rooted out, for the theory of spontaneous generation has long since been destroyed. This result is, unfortunately, beyond the scope of reasonable expectation; but it is not beyond it to hope that its transmission may be limited, for this hope is being realized every day that passes. The question of the communicability of pulmonary phthisis is one that was settled long ago, though a period of doubt followed this. Aristotle states that the contagiousness of consumption was a common belief among the Greeks. Galen regarded it as unwise to pass even a day with a consumptive. Morgagni, as early as 1761, would not make a dissection of a phthisical patient, because of the risk he thought he might run of taking the disease. The question of the communicability of phthisis is no longer a disputed one. The laboratory and the hospital, to say nothing of the hundreds of cases in general practice, all testify to that. I, myself, have carried out the injection of a perfectly healthy rabbit with a pure culture of tubercle bacilli in sterilized water. (Villemin was the first to inoculate animals—in 1865.) The injection was that of a very minute quantity of tubercle bacilli in distilled water into the lung substance of the rabbit. At the autopsy, six weeks later, not only the lungs, but the liver was found studded with tubercular nodules. This is an experiment which can and has been verified time without number. Another form of experimentation was carried out many years ago by an observer who made a number of healthy dogs breathe the atomized sputum of tubercular patients for a certain number of hours every day, and if I mistake not, nine out of the eleven dogs died of phthisis. While such demonstrations from the laboratory of the pathologist ought to be convincing, additional testimony can surely be had from the record book of every active physician. How many faithful and devoted women—wives, mothers, sisters—offer up their lives on this altar of sacrifice. It is also a fact of common observation how many nurses yield to this dread disease.

It has long been a matter of discussion as to whether or not the human fœtus is ever born with phthisis. That is whether or not there is a direct transmission from the father, on one side, through the male element, or the mother, on the other, to the fœtus in utero. The preponderance of evidence seems all against such infection. There is, doubtless, the inherited tendency, weakness, diathesis, or what you will; but that is all, so far as our present knowledge goes. A highly respected authority states that tuberculosis is *not* manifest in the new born. It acts like those rare cases of late congenital syphilis, whose symptoms often do not appear until the second decennium, and whose nature, whether dependent on congenital or post-fœtal infection, is still a matter of controversy. There may be grounds for both positions; but, to me, it seems far more reasonable to conclude, from the facts, that the infection comes through the oft repeated kissing and fondling to which young infants are so often subjected, and also to the fact that mothers and nurses, alike, often use on babies the handkerchiefs and cloths which they themselves use. In this connection, I cannot too strongly urge the obligation resting upon parents and family physicians to not allow the employment of a phthisical nurse.

The intermarriage of tubercular and non-tubercular individuals is an extremely delicate subject for the physician to act upon. While it is highly imperative, from a standpoint of "natural selection," to *prohibit* such unions, it cannot be done in the sacred relations of man and wife. Marriage cannot and should not be reduced to such a basis. It is possible, however, to discourage these unions in a mild way, and to educate the youth of this and coming generations up to the understanding that the highest order of man, physical, intellectual and moral, comes through the union of individuals combining all of these qualities. When marriage has taken place, e. g., between a healthy man and a phthisical woman, it is the physician's duty to explain to the mother the great danger of kissing her child in the mouth. A mother's spirit of self-sacrifice for the good of her child is universally so strong that a little education on this point is all that is necessary. Sir Joseph Clark says "the marriage of consumptive females for the sake of arresting the disease by pregnancy is morally wrong and physically mischievous." The moral and physical wrong are just as great, if not greater, I may add, for a phthisical male to marry, because woman's sense of tenderness and devotion is so heavenly that all laws of health and prudence are lost in her eager desire to minister to the stricken loved one. The reason that infant mortality is so great is that during this stage of development there is a greater demand from the brain and nervous system for nourishment than ever occurs afterward, and this demand must be respected, even at the expense of the other tissues. And since it is estimated that the brain reaches its full size by the fifth year, it is imperative that the body during this period of enormous nervous growth should be especially well nourished; since it is a well known principle in bacteriology that germs will not develop in perfectly healthy serum.

"Ventilation" is a subject upon which the people at large are not well educated. It should be brought more prominently forward and emphasized more strongly than is now the custom in our schools and colleges for young men and women. Every sleeping apartment should have an entrance for pure air, and a means of exit for the foul air, without subjecting the occupants to draughts. For this purpose it is best to have one window, furthest from the bed, lowered slightly from the top, and raised a little way from the bottom. This making a gentle current, the cool fresh air coming in below and the warm vitiated air going out at the top—thus producing a constant cleansing of the atmosphere in the room. The same is true of schools, lecture halls, theaters, asylums, and so forth. There is a general opinion that the sense of oppression and stuffiness, the headaches and sense of malaise produced upon individuals by badly ventilated and crowded halls, is due to the presence of carbonic acid gas ($CO_2$). Such is not the case; it is due to air vitiated with germs and animal particles exhaled from the lungs of those forming the assemblage. $CO_2$ is not poisonous, as is commonly supposed. It is no more a poison than is a physical cutting off of the breath. It is a very heavy gas, and can be poured out just as one would pour a liquid from a vessel. It acts injuriously, in that it displaces the essential element oxygen, and causes death by suffocation. It is the duty of the various Boards of Health to see to it that public halls, schools and prisons should be properly ventilated. On these points the frequency of phthisis among prisoners is especially suggestive. Baer sums up his experience as prison physician as follows: "An intact, i. e., non-tuberculous condition of the lungs in the body of a prisoner is to be regarded as an exception to the rule." This is due not only to the confinement and absence of sunshine in the life of the average prisoner, but to the mental anguish and a relinquishing of all hygienic laws of cleanliness; and the necessity, not only for eating impure, but almost always poorly cooked and unpalatable food. The effects of the chastisement of the body and vigorous disciplining of mind, was shown most remarkably among the nuns in a convent in France, where the religious rites were extremely severe. The ravages made in their numbers was sufficient to cause the greatest comment, and a protest from the attending physicians to the mother superior. Dr. Von Ziemssen, in a recent article, states rather suggestively that: "It has been determined statistically with regard to the French and English armies, that the number of phthisical affections rapidly diminishes with the beginning of war operations and manœuvers, and rises at once with the return of peaceful life in the barracks.

Especial prophylactic precautions are necessary in those whose vital forces are diminished and whose blood is impoverished by any diathesis as e. g., the scrofulous, and after all acute processes such as pneumonia, la grippe, typhoid fever, etc., where the blood is greatly impoverished and the vitality lowered. In all such cases it is essential to avoid all known sources of infection where a healthy individual might go with reasonable safety, and also to carry out hygienic precautions of diet, exercise, etc.

*Sputum.*—Beyond all question, the chief method of infection is through the sputum. It dries on cloths, the floor and every place where it rests, and the bacilli when dried attach themselves to the little atmospheric particles which are carried about by every wind that blows.

The cuspidore, which is an American invention, and which has been a source of so much ridicule by those on the other side of the Atlantic, has turned out to be an article of public good and general adoption. These should be half filled with some disinfecting liquid, and as an additional precaution, the contents burned when emptied. A Russian has made the suggestion of making spitcups of paper, so that they could simply be tossed into the fire after using. What is even better is for phthisical patients to use pieces of cheese cloth (the regular hospital gauze) which is extremely inexpensive, and can be burned after being used. Flies, and mosquitoes, and insects generally, are actively concerned in the transmission of this disease. They swarm around and in the cupsidores and secretions of phthisical patients, and then light upon the noses and mouths of other individuals. This should be avoided as far as possible by the use of wire screens and fly papers in the sick chamber. Another most prolific cause of infection comes in the articles of diet. It is a well known fact that tubercle bacilli are found abundantly in beef, milk of cows, and poultry generally, especially in the flesh of chickens. One cannot but admire the wisdom and foresight of the German meat-inspection laws, which are most stringent. Too much care cannot be taken in the inspection of the meats that come to our tables. The German law does not admit of the sale of any part of a beef until portions thereof have passed in microscopic review before the eyes of an inspector employed by the government. The necessity for such a law in this country is imperative, and it is difficult to understand how our legislators allow this subject to rest as it does when it is known that from one-seventh to one-tenth of the annual death rate of the country is from phthisis. I cannot resist giving here in detail some points of vital interest. Villemen states that "raw or half cooked meats or blood which may contain living germs of tuberculosis should be prohibited, and milk for the same reason should be boiled."

Dr. Dixon, of Philadelphia, Penn., has shown that calves and pigs fed upon milk infected with tubercle bacilli were made tubercular," but milk, I may add, even from tuberculous cows, with tubercle bacilli in it, is rare.

Dr. Henry Behrends, a noted Hebrew physician of London, attributes the great freedom of the Jews as a people from phthisis due to the religious rules concerning the choice and killing of cattle and the sale of meat. He writes that "of 13,116 beeves slaughtered for the Hebrew trade in London in six months, only 6,993 came up to the peculiar Jewish requirements, and that the average rejections for five years had been 40 per cent. But the rejected beeves are often used by Christian butchers." He also states that "in a large practice of over thirty years, he has never met with a case of consumption among the members of the Jewish faith, and that other Hebrew physicians have had a similar experience."

*Sanitary* inspection of slaughter houses and dairies should be enforced. The method of inspection practiced by the Jews, according to Behrends, is as follows: "Kosher meat, as the passed-inspection Hebrew meat is called, is thus prepared under the inspection of the proper official, often the rabbi himself, certainly one familiar with pathological appearances. A perfectly sound and presumably healthy animal is selected and thrown, and a keen sword-like knife, three feet long, is pushed once across the throat and then drawn forcibly back toward the operator, the animal then being hung up by the heels until thoroughly drained of blood. That oft-quoted jugular vein is, of course, severed, and so are the large arteries, by those terrible cuts, for the knife goes to the bone. Every organ is then carefully examined for traces of disease, special attention being paid to the lungs, which must be non-adherent to the chest or to each other in any lobe, and must be fully inflated, then cut into and examined for foci of disease. The larger veins and arteries must then be dissected from the meat, for it is along them that abscesses are generally found, if found at all. If a defect is found in any of these points the meat is rejected as unfit for Jewish use."

Another very important point in the prophylactic treatment is to regulate the time spent in the sick room by relatives, friends and nurses; and to require of these a certain amount of recreation and out of door exercise. A patient is apt to be exacting of a nurse, and especially of a near relative; but the physician should recognize his responsibility and remain firm in his demands. In no instance should wife and husband occupy the same bed if one is infected, and similarly for parent and child.

OCCUPATION.

Individuals with the least hereditary taint, or with any tendency toward pulmonary involvement, should carefully eschew occupations which induce its development, *e. g.*, the baker's trade, where the lungs are constantly irritated by flour dust; or the occupation of needle grinders, stone masons, quarry men, cotton and wool carders, employment in hemp factories, etc.

In large cities, the making of frequent excursions to the country, by the anæmic, scrofulous, and those of the crowded tenement districts suffering from hereditary taints, is of the highest therapeutic value. One of the greatest institutions of New York City is Central Park, where the families of her working districts can go and enjoy God's sunshine, in an all day romp, one day in seven. It is New York's great breathing pore, and a blessing to the city.

CLIMATIC AND HYGIENIC TREATMENT.

The climatic and hygienic treatment of pulmonary phthisis is now being studied more carefully than ever before. The results accruing fully justify all the study put upon this subject, and to this method we may look for much advance to be made in the treatment of phthisis. So far back as the age of Hippocrates do we see the question of climate and its effect on disease considered, for Hippocrates himself says: "Whoever desires to understand medicine thoroughly can by no means neglect the study of the seasons, with their variations of winds, both as to heat and cold, and those peculiar to certain regions, and of the properties of different waters."

Some men infer a connection between dampness of the soil and a prevalence of phthisis, and there are others, strange to say, who believe in sending their patients to the malarial districts, considering malaria, though no one can tell why, as an antidote for phthisis. As a method of prophylaxis, except as a last resort, we would object to this. In this connection let me enter a plea against the *general* advice so often given, to "Go South, young man, or young woman," as the case may be. Now, the South embraces a wide territory, and in this there are localities about as injurious for phthisical patients as it is possible for a place to be. The climatology and topography of the South should be more carefully studied, and individual places prescribed rather than such a broad generalization indulged in. No place or region can be universally popular as a resort for phthisical patients. This for two very good reasons :

1. In a disease of so complex a nature and with such varying complications, it is evident that one locality would be beneficial in some cases and not in others, *e. g.* While it can be said that, as a rule, dry, mountainous regions are good for the general run of phthisical cases, still, in cases of rheumatic or gouty diatheses, feeble circulation, bronchitis, emphysema and albuminuria, mountain altitudes are contra indicated. The rarified air of mountainous regions acts as excitants to coughing, increasing the lung activity, and in the normal individual it has been observed that both adults and children, living in high mountain regions, have relatively larger chest than those living at lower elevations and on the plains.

2. The other reason why no place can become universally popular as a resort for the phthisical is, that the moment a place enters upon a well-earned popularity, numbers of cases hopelessly advanced in the disease flock to it. The large death rate that follows is attributed to the place, and not to the effect naturally resulting from such an influx of patients.

It is believed by many that the tubercle bacilli are practically harmless so long as its dear friends and powerful allies, the streptococus pyogenes, aureus, and albus, are absent. That is to say tubercle bacilli remain harmlessly inert so long as the pus forming element is wanting. Prof. Tyndall's experiments, to determine the merits of the question of spontaneous generation, is of value in this connection. He boiled and filtered a vegetable infusion and hermetically sealed it in flasks, which he transported to the Alps, 7,000 feet above the level of the sea. In the flasks that were exposed to the air at places along the ascent myriads of micro-organisms developed, while that opened at the top remained perfectly free.

In both mountain and sea air we find a larger amount of ozone and greater freedom from organic impurities than in the air of the plains. Dr. Alfred Lee Loomis, who is perhaps the first authority in America on this subject, states that "sea air is better suited than mountain air to those who cannot bear sudden changes of temperature, while the susceptibility to such changes is greatly lessened by mountaiu air." He further adds that "high altitudes are indicated in every stage of fibroid tuberculosis."

Among the mountain resorts that have been tried and not found wanting, may be mentioned East Tennessee and Western North Carolina, especially in and around Asheville, N. C., where Dr. Karl Von Ruck has his sanitarium. Also Denver, Col., and Southern California. Davos Am Platz, in the Swiss Alps, is most highly recommended. It is 5,200 above the sea, is very dry, and not windy or changeable.

Dryness, in itself, is not the only quality desired. The desert of Sahara is as dry a region as can perhaps be found upon the earth, and yet experience teaches that it is one of the worst places that can be found for rheumatic cases. It is due to the fact that persons get intensely heated, and in the first part of the night throw off all covering. During sleep, the radiation of heat from the earth is going on so rapidly, that in the early morning hours the traveler wakes up chilled very much and with stiffened joints. The great value of tenting and camping for phthisical patients cannot be too stongly emphasized. Generally speaking, phthisical patients improve in proportion to the amount of fresh air that they get. It is this point that makes the elevated, sandy regions of the South so efficacious in the cure of phthisis. The climate is so equable, and the number of sunshiny days so great, and the air so benign that the patients are enabled to spend the greater portion of their time out of doors. Localities which correspond, in greater or less degree to these conditions, are St. Augustine, Jacksonville and Tampa, Florida, Aiken, S. C., the Augusta, Ga., sand hills and Thomasville, Ga. Of Aiken, Dr. W. H. Geddings has given much information. In his well written article on the subject, he states that "Aiken is 600 feet above the level of the sea, with a mean temperature of 62.50 degrees, and is on the same isothermal line as Cadiz and Palermo." The sand hills of Augusta are a continuation of those hills upon which Aiken is built, eighteen miles from it, and are only superior to it in that they are connected by a fine electric road, which makes trips to and from Augusta (a city of nearly 50,000 inhabitants, every half hour. Its reputation as a resort for invalids is fast extending over the entire country. Something of its popularity may be gleaned when it is stated that the managers of Hotel Bon Air (which, by the way, is one of the finest and best kept hotels in the South), were obliged to turn away as many as 500 applicants for quarters within the space of one week, this season. The demad for greater accommodations will be yielded to at no distant time. The sand hills constitute an aristocratic suburb of Augusta.—*Concluded in next issue*.

